# Physicians’ perspectives on the treatment of patients with eating disorders in the acute setting

**DOI:** 10.1186/s40337-018-0231-1

**Published:** 2019-01-10

**Authors:** Alexandra R. Davidson, Sarah Braham, Lauren Dasey, Dianne P. Reidlinger

**Affiliations:** 1 Faculty of Health Sciences and Medicine, Bond University, University Drive, Robina, QLD 4226 Australia; 2 Gold Coast Hospital and Health Services, Southport, Australia

**Keywords:** Eating disorders, Inpatients, Physicians, Patient care team, Qualitative, Medical, Decision making

## Abstract

**Background:**

Hospitalisation for an eating disorder is rare, however treatment in the acute medical setting can be a life-saving admission. While the multidisciplinary team delivers overall patient care, medical decisions are the responsibility of the treating physicians. Treatment decisions directly impact on patient care and outcomes. This study aimed to explore the considerations that influence the medical decisions of physicians when treating patients with eating disorders in the acute setting.

**Method:**

Semi-structured interviews were conducted with ten medical physicians who had previously treated eating disorders on a general medical unit in two Australian tertiary hospitals. An interview schedule, based on the literature and four relevant domains from the Consolidated Framework for Implementation Research, was developed. Interviews were audio recorded, transcribed verbatim and analyzed thematically. Coding and interim themes and sub-themes were developed by two dietitian researchers; these were further refined through researcher discussion and triangulation with two additional dietitian researchers.

**Results:**

Ten doctors were interviewed (3 consultants (1 adult general medical and 2 paediatricians: 13–16 years medical experience), 2 registrars (4–7 years experience), 1 resident (1 year experience), and 4 interns (< 1 year experience). Doctors described memorable patient cases, related to hospital stays over several weeks. Interviews ranged in length from 58 min to 91 min. Four themes (*with five sub-themes*) were developed: 1) navigating uncertainty (*focusing on processes and goals and seeking information*), 2) being “the good doctor” (*doing the right thing),* 3) seeing the big picture (*depending on key players and considering short and long-term*), and 4) involving family and patient.

**Conclusions:**

Non-specialist physicians described challenges in the treatment of eating disorders in the inpatient setting. They take a holistic approach that considers both short and longer-term goals, relying on specialist colleagues, the wider multidisciplinary team and sometimes family members to guide treatment decisions during admissions on general medical wards. Additional support, education and training centered on the key themes may increase physicians’ confidence and ability to make effective treatment decisions for this patient group. The results are relevant to all health professionals working in this field to better understand the priorities of medical physicians and to support them to achieve positive outcomes in the inpatient treatment of patients with eating disorders.

**Electronic supplementary material:**

The online version of this article (10.1186/s40337-018-0231-1) contains supplementary material, which is available to authorized users.

## Plain English summary

Hospital treatment of eating disorders can be challenging for both patients and health care professionals. Physicians were of interest in this study, as they are responsible for the medical decisions made in the treatment of patients with eating disorders during a hospital admission. Typically, these patients are admitted to general medical wards, rather than specialist psychiatric wards. This research sets out to understand physicians’ experiences when caring for patients admitted to hospital for urgent, often life-saving, treatment. Ten physicians were interviewed about their experiences with eating disorder patients. Key themes were developed to explain the underlying reasons for physicians’ decisions in the treatment of these patients, their challenges and how they overcome these. Four themes (and five sub-themes) were 1) navigating uncertainty (focusing on processes and goals and seeking information), 2) being “the good doctor” (doing the right thing), 3) seeing the big picture (depending on key players and considering short and long-term), and 4) involving family and patient. Physicians relied on family members, specialist colleagues, nurses, dietitians and other allied health professionals to ensure safe, evidence-based treatment that was focused on both short and longer-term recovery goals.

## Background

Eating disorders are characterized by ongoing abnormal and distorted eating habits or behaviours [1]. It is estimated that 30 million people in the USA, 1.25 million people in the United Kingdom and 1 million people in Australia, are affected by an eating disorder [2–4]. Eating disorders have the highest suicide and co-morbid mental illness rate of all mental health illnesses and are predominately managed in the community [5, 6]. Although only a minority of people with an eating disorder are hospitalized [7], acute medical treatment is focused on medical stabilization, which can be lifesaving, and an important part of an individual’s recovery. The most common diagnoses to require admission for medical stabilization are Anorexia Nervosa and other restrictive eating disorders [8]. Such treatment may include electrolyte replacement, monitoring and correction of refeeding syndrome, electrocardiograph monitoring, and oral or tube feeding [9, 10].

Caring for this patient group within the acute medical setting is known to be challenging [11]. There is a need for a cohesive approach encompassing medical and psychological management, and to manage the patient’s ambivalence towards medical intervention [11]. A multidisciplinary team approach and the use of guidelines can facilitate treatment and overcome challenges [12]. A range of guidelines for the treatment of people with eating disorders across all settings are available to ensure optimal and consistent care of patients. In the United Kingdom, the National Institute for Health and Clinical Excellence has recently developed new guidelines for recognition and treatment of eating disorders, including in the acute environment [13]. Clinical practice guidelines from the American Psychiatric Association cover all eating disorder diagnoses [10]; and in Australia, there are a range of similar clinical guidelines including those produced by the Queensland Eating Disorder Outreach Service [14]. The aim of these guidelines is to provide health professionals with a framework for daily medical measurements and behavioural management techniques for patients [14]. However, the uptake of guidelines is known to be inconsistent and dependent on a range of factors including awareness and effective implementation [15].

The experiences of nurses, dietitians, psychiatrists and primary care physicians working with patients with eating disorders have previously been explored and assessed across quantitative and qualitative studies [16, 17]. However, hospital physicians – that is, those medical professionals working in general medical rather than specialist psychiatric or mental health wards - have not previously been studied as a homogenous sample. Given the challenges highlighted across other health profession groups, and the key role of physicians in acute medical wards in managing these patients, further insight into the reasons for treatment decisions in this patient group, including those which may diverge from guidelines, is warranted [18]. For other health professionals, a better understanding of the considerations informing the treatment decisions of physicians could support a more team focused approach to the care of patients with eating disorders admitted to non-specialist wards. This study aimed to explore the considerations that influence the medical decisions of physicians when treating patients with eating disorders in the acute setting.

## Methods

This was an interview study targeting physicians involved in the treatment of eating disorders in two acute metropolitan hospitals in South East Queensland, Australia. Interviews were chosen as the most appropriate method for exploring their experiences with eating disorder patients, in order to better understand the treatment decisions made by individual physicians, whilst providing a safe space to express potentially sensitive information about their challenges and areas of uncertainty in treating this patient group. An interview schedule (Additional file 1: Table S1) was developed by the research team to elicit in-depth responses from the physicians to inform the research aim. Questions were developed based on the literature and according to four relevant domains from the Consolidated Framework For Implementation Research (CFIR): *(1) characteristics of the individuals involved, (2) process, (3) outer setting, and (4) intervention characteristics* [19]. To provide richness of data through real case vignettes, physicians were asked to recall and describe two patient cases they had previously treated: 1) a patient with an eating disorder treated voluntarily and 2) another patient treated involuntarily [20]. Prior to data collection, a pilot interview was conducted with a medical intern, not eligible to participate, to refine the interview schedule.

A convenience sample was sought, due to the known difficulties in recruitment of physicians to research studies [21]. All eligible physicians who were willing and available were invited to participate. Physicians from all experience levels currently working at the hospitals were eligible, provided they had previously treated a patient with an eating disorder in an acute medical setting (such as a general medical ward admission). Medical students, nursing and allied health staff were excluded. One researcher recruited eligible physicians in a brief information session at medical handover meetings and teaching sessions at both hospitals. Recruitment snowballing was used: once physicians had participated, they were then asked to pass on the study information to potentially eligible colleagues. Signed and informed consent was obtained before each interview.

Face-to-face semi-structured interviews were conducted by one researcher in a private room at either hospital site. Interviews were audio recorded using a digital voice recorder and transcribed verbatim by one researcher and checked by a second researcher against the recordings for accuracy and to facilitate immersion in the data [22]. To ensure anonymity and confidentiality, potentially identifiable data disclosed during the interviews were removed during transcription and replaced with pseudonyms, including details of names, locations, and patient details. Inductive, line-by-line analysis of data was undertaken immediately following transcription of each interview. The interviewer recorded field notes after each interview to aid the analysis, and data collection ceased when there was consensus among two of the researchers that the study’s aim had been addressed. Thematic analysis followed a five-step process as recommended by Braun and Clarke: 1) data familiarization, 2) initial coding, 3) theme development 4) theme review (whereby preliminary themes were applied to all codes until theoretical sufficiency was reached) [23] and 5) theme defining and naming [24]. Microsoft Word was used to manage the data. Researcher triangulation ensured trustworthiness during thematic analysis: coding and preliminary themes were developed, discussed at length and agreed between two researchers, the selected exemplar quotes were then presented and discussed until there was agreement on the final themes and sub-themes amongst all four researchers (three of whom were dietitians, and the fourth of whom was a dietetic student). The manuscript was prepared according to the RATS qualitative research review guidelines [25] (Additional file 2: Table S2). Ethical approval was obtained from the (Bond University Human) Research Ethics Committee prior to commencing recruitment, Ref: 015660.

### Findings

Eleven physicians were recruited, ten were interviewed. One physician provided written informed consent, but was deemed ineligible to participate due to having no experience of treating eating disorders in an acute medical ward. Participating physicians had a range of medical experience; three attending physicians (one adult general medical and two pediatricians: 13–16 years medical experience), two fellows (4 to 7 years medical experience), one junior resident (>1 year medical experience), and four interns (<1 year medical experience). During the interviews, participants chose patient cases to describe in detail, in response to the questions posed in the interview schedule. The majority of physicians described experiences related to people with anorexia nervosa; one participant described their experience with a patient with bulimia nervosa.

**Fig. 1:**
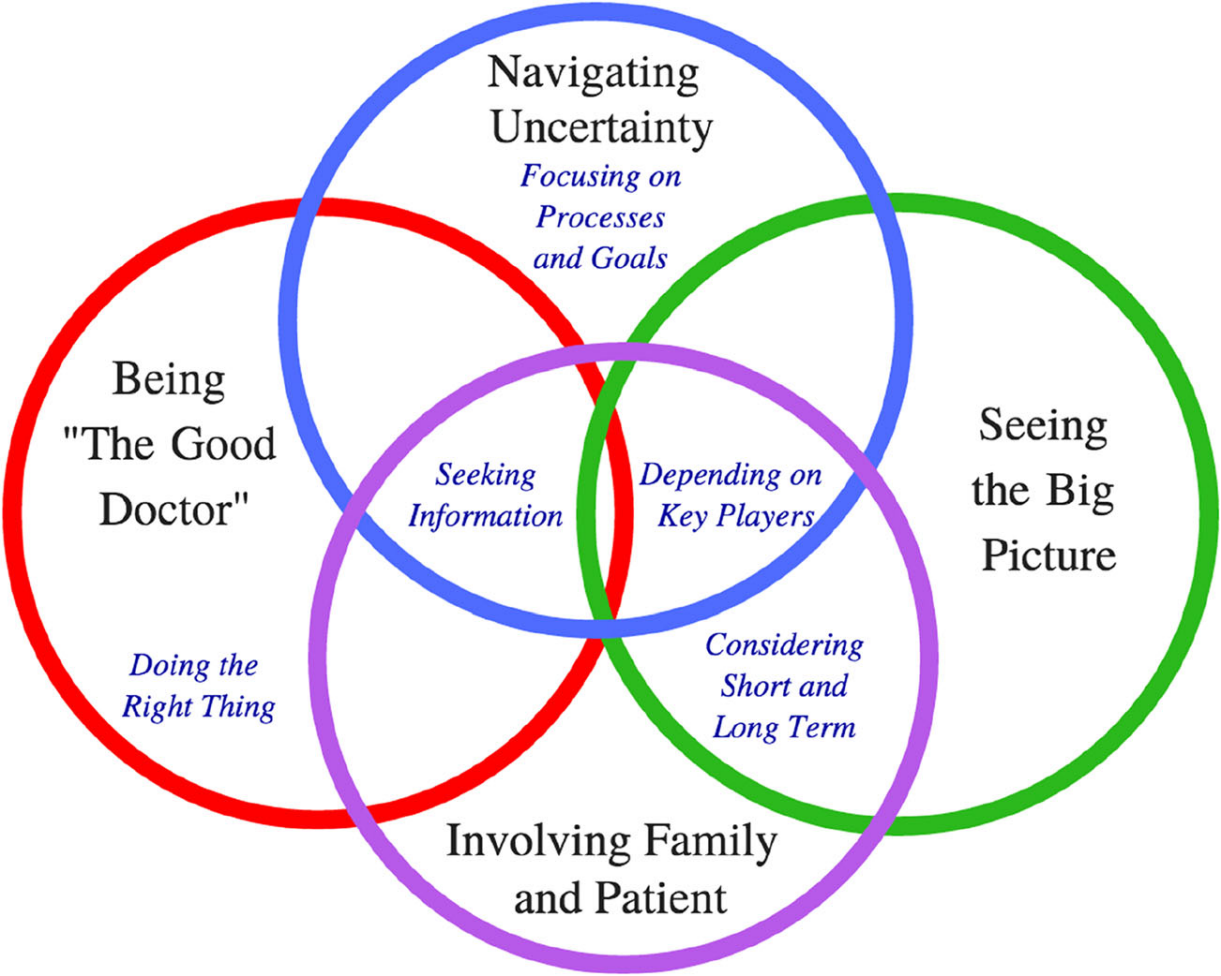
Summary of themes and sub-themes and their interrelationships

Four themes (*navigating uncertainty, being the good doctor, seeing the big picture, involving patient and family*) and five sub-themes (*focusing on processes and goals, seeking information, depending on key players, considering short and long term, and doing the right thing*) were developed (Fig. 1).

### Theme 1: navigating uncertainty

Within this theme, three sub-themes were developed: 1) focusing on processes and goals, 2) depending on key players and 3) seeking information. Physicians described the treatment of patients with eating disorders as outside of their comfort zone. When describing cases, physicians were more likely to express confidence about the objective medical tests and interventions, but were less confident communicating with the patient and behavioural management. The physicians working in adult wards related this uncertainty to the relatively rare experience of treating patients with eating disorders.

*“…they’re very few and far in between…So, it doesn’t happen often for us.” – Physician #1.* (male, attending physician, 14 years medical experience).

Whilst physicians working in pediatric wards did not comment on the rarity of eating disorder patients, they still described the challenges in navigating this uncertainty:

*“Sometimes I do wonder whether the way I approach things or whether the way I say certain things, whether that’s actually the best way or not.” – Physician #10* (female, pediatrician, 13 years medical experience)

Physicians, from adult and pediatric units, described their uncertainty about management of eating disorders as due to eating disorders not being “black and white” - the decisions they faced, in particular behavioural management decisions, were subjective. Physicians described how they would navigate this uncertainty by seeking information: for example, referring to a guideline or protocol, the evidenced-based literature, and other health professionals from the multidisciplinary team whom they identified were key players in effective treatment, including specialist colleagues in the liaison psychiatric services, dietitians, psychologists and nurses.

*“…talking to my other consultant colleagues, especially with regard to approaching family and conflicts between medical staff and family and how they’ve managed them, influences in a way cause they’ve done it more often than me.” – Physician #1* (male, attending physician, 14 years medical experience – referring to liaison psychiatric services colleagues in the hospital).

### Theme 2: being “the good doctor”

Within this theme there were two sub-themes: 1) doing the right thing and 2) seeking information. Physicians described that fulfilling “the good doctor” role was an internal drive to do what is best for their patient and patient safety. The elimination of the cause of the patient’s unsafe medical status was the physicians’ main priority in treatment:

*“…obviously for a patient that’s in the acute setting, you have to be worried about refeeding syndrome. And you wanna do that safely.” – Physician #8* (female, intern, less than one year medical experience).

Physicians described their responsibility to do the right thing by the patient, the family and other treating health professionals. This included meeting the expectations of the patient and the family, and answering complex questions proposed by family:

“*It’s difficult because they have so many straight, closed questions that they want answers to that never have straight answers…But, I feel like we don’t always tell them what they expect to hear…”- Physician #2* (male, junior resident, 1 year medical experience).

However, there was one deviant case, a general medical attending physician, who described how he would only focus on what he believed was right for the patient, even if this meant ignoring the family’s demands and protests, which in some cases was difficult to do:

*“…the main thing that makes me determine what to do with a patient is what’s going to be best for them and not what’s best for the family, or for anyone else.” – Physician #1.* (male, attending physician, 14 years medical experience).

Physicians described that being “a good doctor” also included being a life-long learner. Treatment of eating disorders was described as being only ‘briefly touched on’ in medical school, thus physicians often identified eating disorder issues as something of a gap in their knowledge and experience. During medical school, physicians described that the importance of seeking information from credible sources to guide practice was instilled in them:

*“...if, you know, we’re ordering an investigation or something and I don’t really understand why, if I don’t get the opportunity to ask someone more senior then I generally like to sort of look into that myself…”* – Physician #6 (intern, female, <1 year experience).

### Theme 3: seeing the big picture

Within this theme were two sub-themes, 1) depending on key players and 2) considering short and long-term. In this theme, physicians spoke of the importance of having an overview of all aspects of care as vital in the patient’s treatment. Physicians described that from admission, through to transfer (for example to an inpatient psychiatric unit within the hospital) and discharge, considering the patient’s condition from multiple angles, not just a single variable such as body mass index, assisted in their decision-making:

*“I don’t think [admission] is always just a number... it’s always a picture of things.” – Physician #2.* (male, junior resident, 1 year medical experience).

Physicians described this overarching approach included short and long-term goals, but also included collaboration with a range of health professionals from the multidisciplinary team. Physicians universally agreed that they could not treat a patient with an eating disorder alone. For their patients with eating disorders they made referrals to specialists to assist with treatment, including a psychiatrist and dietitian. The physicians demonstrated respect towards the other disciplines, so long as these other health professionals fulfilled their role in treatment.

The sub-theme “short and long-term” encompassed all aspects of treatment. Physicians detailed that their patients typically spend several weeks on a general medical ward, reflecting the longer length of stay for this patient group (a mean of almost 20 days compared to 2.9 days for other admissions in Australia) [7]. They described their role in medical stabilization of the patient as only a temporary fix, acknowledging that if discharge was not followed up with long-term care then the patient would not recover. To ensure long-term care was applied one physician described how this influenced her discharge planning for patients:

*“…my goals are generally short-term goals, so I wanna make them safe enough. My long-term goals with these patients is to provide them with a plan and then a support network which would usually include an outpatient psychologist and dietitian.”* – Physician #5 (female, fellow, 7 years medical experience).

Re-admissions were expressed as one of the many challenges to treating this patient group because physicians then felt that their own previous treatment attempts had failed. One physician felt that the reason for re-admissions was because time between discharge and follow-up appointments in the community was too long, and many patients were being re-admitted to hospital in that time:

*“So, you discharge someone, cool, we’ve got you to a good BMI. They start to go yeah; okay I think we might win this one… your outpatient appointment’s in two weeks or three weeks with psychology... In that two weeks they’re back in again.” – Physician #2.* (male, junior resident, 1 year medical experience).

### Theme 4: involving family and patient

This theme encompassed two components: firstly, the involvement of family in treatment and decision-making. Physicians working on pediatric wards described that involving the patient’s parents was mandatory. Physicians treating adults described that the family was only involved in treatment with the patient’s consent. However, both described that family could be a ‘double-edged sword’. On one-hand family was identified as a useful tool in treatment, such as admitting the patient to hospital, and their role in family-based therapies. When family were perceived as supportive and able to contribute to treatment, physicians readily involved them:

*“…they’re going to be the ones to keep an eye out for warning signs. So, it’s important to involve them in their care as well.”* – Physician #5 (female, fellow, 7 years medical experience).

On the other hand, physicians described that family could also be detrimental to treatment. Physicians empathized with the family, and perceived the family had the patient’s “best interest at heart” yet were not always able to express this constructively. Physicians described experiences of family members who were hostile towards the diagnosis and treatment plans, but at times seemed to begrudgingly welcome them into treatment decisions.

“*…So I think it’s…more important… than your regular run of the mill medical patient to be a bit more careful about the family…They can be really supportive and really helpful. But at the same time they can be quite detrimental too.”* – Physician #3. (male, intern, less than one year medical experience).

The second component to this theme was involving the patient. How the physicians involved the patient in treatment was highly variable. Physicians described the patient’s capacity to make their own treatment decisions as more likely if patient decisions aligned with those of the doctor. From the physicians’ perspective patients were generally expected to comply with treatment. However, in the case of eating disorders, the physicians also described anticipation of “non-compliance”. They described treatment of patients with eating disorders as easier if the patient had insight into their illness, where insight referred to a patient’s perception and knowledge of their mental health illness. The physicians described the patient’s insight, or lack of insight, would contribute to the uncertainty and challenges of treatment:

*“…it can be challenging with the patients who don’t have the insight into their illness, because they don’t necessarily want your help, that can be a challenge.” – Physician #4* (male, intern, less than year medical experience).

## Discussion

This study set out to explore the considerations of physicians in their treatment of patients with eating disorders, focused on those that influence medical decisions during acute hospital admissions. Key findings were captured in the four themes and five subthemes developed, which highlighted the uncertainties in treatment decisions, beneficence of physicians in wanting to do what was best for their patients, with an understanding that this requires the physicians to think beyond the immediate acute problems. Physicians identified that support in the form of treatment guidelines and evidence, along with the advice of more experienced colleagues and the wider team were critical to their treatment decisions. They described key challenges they faced due to the relative rarity of eating disorder admissions, the need to tailor the treatment according to the patient and their level of insight, and the key role of family as potential enablers of successful treatment. These findings are relevant to all health professionals working in this field to better understand the priorities of acute care physicians and to support them in their treatment of patients with eating disorders.

The physicians in this study identified patients with eating disorders as unusual, describing the relative rarity of admissions as a key contributor to their uncertainty when making treatment decisions. This perception of ‘rarity’ appears to be real, with the majority of treatment conducted in outpatient and community settings rather than inpatient admissions [7]. The observation that people with eating disorders present for treatment in small numbers is consistent with results from a qualitative study in the UK, which explored the experiences of a range of health professionals providing inpatient and outpatient care for people with eating disorders. This study also found that participants working on a general medical ward felt they were lacking the experience to effectively treat people with eating disorders due to low admission rates [6]. Similarly, the same researchers interviewed general practitioners who described this patient group as small in number, but high in complexity [6].

The finding that physicians rely on practice guidelines to manage their uncertainty when making treatment decisions is perhaps not surprising. A guideline for the inpatient medical management of a patient with an eating disorder is available to physicians working in the adult and pediatric wards within the health district studied [14, 26] although it was clear during the interviews that different physicians used different guidelines, and some were unaware of the guideline. Some doctors relied on more senior colleagues and/or other health professionals to guide their treatment decisions, rather than the guideline. Protocols and guidelines aim to support health professionals in facilitating treatment to overcome potential challenges with eating disorders [14]. They have been identified as useful for doctors and other health professionals to overcome challenges and barriers, such as communication and the complexity of individualized management in patients with eating disorders in the acute medical setting [27]. Additionally, it has been suggested that the less experienced the health professional is in treating a condition, the greater the reliance on guidelines in treatment [15]. However, physicians in the present study expressed a reliance on guidelines regardless of their experience level.

The commitment to lifelong learning by physicians in this study reflect professional expectations of continuing professional development and evidence-based medicine [28, 29]. Knowledge of eating disorders and of appropriate acute medical treatment is vital for physicians to effectively manage patients with eating disorders. Research has shown that insufficient knowledge of eating disorders can result in lowered confidence in treating patients, which is consistent with our findings [18, 29]. Opportunities for professional development in effective treatment and relevant guidelines for this patient group may go some way to meeting the needs of physicians in the acute setting, perhaps in an interdisciplinary setting to encourage sharing and understanding within the multidisciplinary team.

The beneficence of physicians in this study was highlighted by their motivation to be the “good doctor”. This likely reflects the altruistic nature of those that choose medicine as a career, along with the professional and ethical conduct codes imbued in medical school training and upheld by physicians in their practice [28]. The responsibility to do what is right by the patient, and to meet expectations placed on the physicians by both patient and parent (family) has been previously described. Health professionals interviewed in a UK study expressed intentions to do what is best by their eating disorder patients, but noted uncertainty about the correct course of action for their individual patients [6]. The influence of perceived patient and family expectations of the physician during inpatient treatment was also reported in an interview study of health professionals working in an adult eating disorder inpatient service, where participants felt it was difficult to meet high expectations of care and the recovery process [11].

The physicians in this study were grateful for the input of other health professionals including mental health specialists to inform their treatment decisions, and supports practice guidelines and literature which advocate multidisciplinary team working as the most effective approach to treatment [17, 30]. Key members of the multidisciplinary team for eating disorders include dietitians, psychologists and medical practitioners [14]. Although each individual specialist has a crucial role in eating disorder treatment, the ineffectiveness of isolated treatment has been noted [13, 31]. These findings should strengthen the role of health professionals working with physicians during acute care admissions.

The physicians in this study described readmissions as setbacks, and attributed these in part to untimely follow up of patients after discharge. Patients with eating disorders have high rates of relapse and readmission [11]. Delayed follow-up has also been described as a problem in the primary care setting, where general practitioners described lengthy referral waiting lists [32]. Ensuring timely and intense follow-up treatment has been identified as one method of decreasing rates of relapse and readmissions [30]; other suggestions include training, primary care referrals and continuity of care as key components of effective care in the primary care setting [6]. Our findings indicate physicians’ frustration and erosion of confidence in the treatment of eating disorder patients as a result of this readmission cycle.

The complexity of care of people with eating disorders extended to the patient and their insights, and the role that the family played in effective treatment. Socially acceptable behaviour for a patient is usually to seek and accept help from a health professional [33]. Eating disorder patients typically go against this norm - poor insight, ambivalence and refusal of professional help are key characteristics of patients with eating disorders, in particular those with Anorexia Nervosa [30]. Other health professionals have noted the propensity for patients to refuse treatment options as complicating treatment decisions in eating disorder care, and described the difficulty of having to treat someone who refuses care [34].

Involving family as a partner in discussions and treatment of a patient is the crux of patient- and family- centered care [35]. Family members are also a valuable source of information and play an important part in the long-term care of the patient. The hesitation to involve family was also previously described by health professionals who worked with children and adolescents with eating disorders in a range of settings. Participants in the current study found the decision to involve family as more difficult than the objective medical decisions [36]. Support for physicians to optimize communication with patients and family, possibly through training or professional mentoring, may result in more inclusive treatment decisions that support acute recovery and effective discharge.

### Strengths and limitations

A key strength of this study was the successful recruitment of physicians across two hospitals in the same health district. Physicians are known to be a difficult population to recruit for research due to many reasons, including demanding schedules, which occurred in this study [37]. The decision to only include general medical physicians meant an initial, smaller pool to recruit from, but also provided a unique homogenous participant group who represent key decision makers in the medical treatment of eating disorder patients admitted to hospital. The information gathered from physicians who participated in this study provided a range of experience and insight into the research question. With this smaller sample size, theoretical sufficiency (whereby new data was able to be accommodated within the developed themes without further modifications) was achieved [23], although it is possible that with a larger sample size, a greater number or modified themes may have been developed. However, interviews were adequate in length, about 1 h each – remarkable considering the busy schedules of the participants - and produced rich data into the experiences and influences of the physicians involved. The patient cases described by physicians were chosen by them, and it is possible that the cases described did not reflect the full gamut of admissions to a general medical ward such as short admissions for electrolyte correction only. In addition, the descriptions were limited to details that the doctor could recall during the interview, and in some instances, these may have been unclear or misremembered. For example, some physicians could not recall whether treatment was involuntary or voluntary, but nevertheless chose a case because they were comfortable to discuss it in detail. Analysis was conducted by researchers who were all dietitians or a student dietitian, and we acknowledge that this almost certainly influenced the final themes developed and presented here; analysis by researchers with a more diverse professional background may have resulted in different interpretations of the data. The analysis adds insight into the thoughts and concerns of treating physicians, we believe for the first time.

The study findings highlight future opportunities for research and service improvements in this area. They emphasize the need to build physicians’ (and medical students’) knowledge and confidence in working with patients with eating disorders. Training for physicians, and perhaps also allied health colleagues supporting them, that is focused on awareness of existing resources, including guidelines and protocols for inpatient treatment, and skills to manage the challenges of working with patients with eating disorders, their families and colleagues is warranted. Conducting a similar study with other health professionals involved in the treatment of eating disorders in the acute setting, such as nurses, dietitians and psychologists, could add to the more effective implementation of guidelines within acute medical wards.

## Conclusions

The physicians viewed the multidisciplinary team approach as vital to the treatment of eating disorders in an acute setting, which should instill confidence in other health professionals working in this clinical area. Further, their awareness of the physicians’ experiences, including an understanding of the challenges physicians face with this patient group, should ensure other members of the multidisciplinary team can better support the medical team to more effectively treat them in the acute setting. The results are relevant to all health professionals working in this field to better understand the priorities of acute care physicians and to support them in their treatment of patients with eating disorders. Such awareness is likely to improve team dynamics as well as outcomes for this patient group.

## Additional files

Additional file 1: **Table S1.** Interview guide with rationale for inclusion. (DOCX 31 kb)

Additional file 2: **Table S2.** Qualitative research review guidelines – RATS. (DOCX 23 kb)
