# Supplementary material for: Physicians’ perspectives on the treatment of patients with eating disorders in the acute setting
Source: J Eat Disord. 2019 Jan 10;7:1. doi: 10.1186/s40337-018-0231-1 (PMC6327410; doi:10.1186/s40337-018-0231-1)
Supplement: Supplementary file 1 — Table S1. Interview guide with rationale for inclusion. (DOCX 31 kb) [file 40337_2018_231_MOESM1_ESM.docx]

| Semi-structured Interview Guidance - Tool – Version 5 | | | |
| --- | --- | --- | --- |
| **No.** | **Type** | **Key question and prompts** | **Justification from literature, or research team discussion** |
| 1 | Intro / Ice breaker  Domain^1^: Characteristics of Individuals  Construct: Individual identification with organisation | 🡪 Can you tell me about your previous experiences caring for patients with eating disorders?  PROMPTS: In the acute care setting here, and in other areas? Have you had experience at/in any other setting? | Tong, et. al. 2014^2^ |
| 2 | Case Questions  Domain^1^: Intervention Characteristics  Constructs: Intervention source, adaptability, complexity.  Domain: Outer Setting  Constructs: Patient needs Cu and resources | 🡪 Could you tell me about a specific time you were involved in the treatment of a patient with an eating disorder, who ***was*** receiving treatment voluntarily, (without an ITO), in an acute setting, even if this was just for part of their admission?  PROMPTS: Treatment plan? Who involved in their care? Who were the decision makers regarding treatment or interventions? What was the outcome for the patient? Can you recall what their discharge destination was? What was their health status upon discharge? i.e. BMI Any challenges in the admission, how did you overcome these? How satisfied were you with the outcome/ why? | Research Team Discussion  References re use of vignettes and case study:  Currin, Waller & Schmidt, 2009^3^ |
| 3 | Case Questions  (as above) | 🡪 Could you tell me about a specific time you were involved in the treatment of a patient with an eating disorder, who was receiving treatment under an ITO (involuntary treatment order), in an acute setting, even if this was just for part of their admission?  PROMPTS: Treatment plan? Who involved in their care? Who were the decision makers regarding treatment or interventions? What was the outcome for the patient? Can you recall what their discharge destination was? What was their health status upon discharge? i.e. BMI Any challenges in the admission, how did you overcome these? How satisfied were you with the outcome/ why? | Research Team Discussion  (as above) |
| 4 | Prior experience, training and education  Domain^1^: Characteristics of Individuals  Construct: Knowledge and beliefs about the intervention & Self-efficacy | 🡪What previous experiences or training specific to treatment of eating disorders, have you received? (At any stage i.e. as a student or practising)  PROMPTS: How did you obtain this knowledge or education? For example: experience with a specialist eating disorder unit, university, additional professional development workshops, conferences and courses. Has the training you received made you more confident in treating this patient group? What further training would help with your confidence, or improve your confidence in this area? | Reid, et. al. 2010^4^ Damschroder, et al. 2009^1^ |
| 5 | Decision-Making  Domain^1^: Characteristics of Individuals  Construct: Knowledge and beliefs about the intervention | 🡪 What influences your treatment decision-making, with patients like the ones you just described?  PROMPTS: How does your prior training and knowledge influence your practice? What other factors influence your decisions in treatment? E.g. previous patient experiences, personal beliefs, personal experiences, other clinicians (i.e. dietitians, nurses, etc.), (family, friend), patient requests or goals. | Tong, et. al. 2014^2^ |
| 6 | Family involvement | 🡪 Could you describe to me, how you would interact with a patient’s family?  PROMPTS: Would you involve them in decision-making? | Raveneau, et. al. 2014^5^ |
| 7 | Patient needs and treatment  Domain: Outer setting  Construct: Patient needs and resources | 🡪 What do you understand to be the primary focus of an inpatient stay for eating disorder patients in the acute setting?  PROMPTS: What do you perceive to be the needs of these patients? How do you perceive this differs from the focus of outpatient care? | Damschroder, et al. 2009^1^ |

| 8 | Individual beliefs  Domain^1^: Characteristics of Individuals  Construct: Knowledge and beliefs about the intervention | 🡪 What are your beliefs and/or opinions on treating patients with eating disorders as inpatients in the acute setting?  PROMPTS: This could relate to any aspect of inpatient therapy (e.g what are your thoughts/perspective on treatments such as nasogastric feeding, 1:1 nurse special, strict bed rest, patient’s being treated under ITO, the need for hospitalisation at all, patients being treated on a general medical unit rather than an ED specific unit? | Raveneau, et. al. 2014^5^ |
| --- | --- | --- | --- |
| 8 | Patient behaviour | 🡪 In some cases, patients with eating disorders have been described as “manipulative, is this something you have experienced?  PROMPTS: Can you describe what makes their behaviour “manipulative”? Were/ how were treatment decisions influenced by this behaviour? | Ramjan, 2004^6^  Ryan, et. al. 2006^7^ |
| 9 | Participant opportunity to provide further insight into treatment options | 🡪 Are there any other aspects of inpatient therapy options that you think are essential to their care?  PROMPT: Laminated sheet with aspects of inpatient care to elicit their opinions on each point. |  |

**Closing** – Do you have any other comments or thoughts that could contribute to the study?

References

1. Damschroder LJ, Aron DC, Keith RE, Kirsh SR, Alexander 519 JA, Lowery JC: Fostering implementation of health services research findings into practice: a consolidated framework for advancing implementation science. Implement Sci 2009, 4.
2. Tong A, Mahady SE, Craig JC, Lau G, Peduto AJ, Loy C. Radiologists’ perspectives about evidence-based medicine and their clinical practice: a semistructured interview study. BMJ Open. 2014;4(12).
3. Currin L, Waller G, Schmidt U. Primary care physicians' knowledge of and attitudes toward the eating disorders: do they affect clinical actions? The International journal of eating disorders. 2009;42(5):453-8.
4. Reid M, Williams S, Burr J. Perspectives on eating disorders and service provision: A qualitative study of healthcare professionals. European Eating Disorders Review. 2010;18(5):390-8.
5. Raveneau G, Feinstein R, Rosen LM, Fisher M. Attitudes and knowledge levels of nurses and residents caring for adolescents with an eating disorder. International journal of adolescent medicine and health. 2014;26(1):131-6
6. Ramjan LM. Nurses and the ‘therapeutic relationship’: caring for adolescents with anorexia nervosa. Journal of Advanced Nursing. 2004;45(5):495-503
7. Ryan V, Malson H, Clarke S, Anderson G, Kohn M. Discursive constructions of ‘eating disorders nursing’: an analysis of nurses' accounts of nursing eating disorder patients. European Eating Disorders Review. 2006;14(2):125-35.
