# Supplementary material for: Physicians’ perspectives on the treatment of patients with eating disorders in the acute setting
Source: J Eat Disord. 2019 Jan 10;7:1. doi: 10.1186/s40337-018-0231-1 (PMC6327410; doi:10.1186/s40337-018-0231-1)
Supplement: Supplementary file 2 — Table S2. Qualitative research review guidelines – RATS. (DOCX 23 kb) [file 40337_2018_231_MOESM2_ESM.docx]

Table S2 Qualitative research review guidelines – RATS

| **ASK THIS OF THE MANUSCRIPT** | **THIS SHOULD BE INCLUDED IN THE MANUSCRIPT** | **Line number (page)** |
| --- | --- | --- |
| **R Relevance of study question** | | |
| Is the research question interesting?  Is the research question relevant to clinical practice, public health, or policy? | Research question explicitly stated  Research question justified and linked to the existing knowledge base (empirical research, theory, policy) | Set out as an aim:  Page 4, Line 91-92 Page 4, Line 82 - 91 |
| **A Appropriateness of qualitative method** | | |
| Is qualitative methodology the best approach for the study aims?   - *Interviews:* experience, perceptions, behaviour, practice, process - *Focus groups:* group dynamics, convenience, non-sensitive topics - *Ethnography:* culture, organizational behaviour, interaction - *Textual analysis:* documents, art, representations, conversations | Study design described and justified i.e., why was a particular method (e.g., interviews) chosen? | Page 4, Line 96-100 |
| **T Transparency of procedures**  **Sampling** | | |
| Are the participants selected the most appropriate to provide access to the type of knowledge sought by the study?  Is the sampling strategy appropriate? | Criteria for selecting the study sample justified and explained   - *theoretical:* based on preconceived or emergent theory - *purposive:* diversity of opinion - *volunteer:* feasibility, hard-to-reach groups | Page 2, Line 39-40  Page 5, Line 111-112 |
| *Recruitment* | | |
| Was recruitment conducted using appropriate methods? | Details of how recruitment was conducted and by whom | Page 5, Line 115-119 |
| Is the sampling strategy appropriate? |  |  |
| Could there be selection bias? | Details of who chose not to participate and why | N/A |
| *Data collection* | | |
| Was collection of data systematic and comprehensive? | Method(s) outlined and examples given (e.g., interview questions) | Page4-5, Line 100-105, 105 – 109 and 122 - 125  Supplementary Table 1 – Interview Guide |
| Are characteristics of the study group and setting clear? | Study group and setting clearly described | Page 5, Line 113-115 and 122-123 |
| Why and when was data collection stopped, and is this reasonable? | End of data collection justified and described | Page 16, Line 394-395 and Page 17, Line 400-405 |
| *Role of researchers* | | |
| Is the researcher(s) appropriate? How might they bias (good and bad) the conduct of the study and results? | Do the researchers occupy dual roles (clinician and researcher)? Are the ethics of this discussed? Do the researcher(s) critically examine their own influence on the formulation of the research question, data collection, and interpretation? | Page 6, Line 136-137  Page 17, Line 405-408  Page 19, Line 449 and Line 463 – 467 |
| *Ethics* | | |
| Was informed consent sought and granted? | Informed consent process explicitly and clearly detailed | Page 5, Line 119-120 Page 18, Line 437-439 |
| Were participants’ anonymity and confidentiality ensured? | Anonymity and confidentiality discussed | Page 5/6, Line 125-128 |
| Was approval from an appropriate ethics committee received? | Ethics approval cited | Page 6, Line 139-141 Page 18, Line 436-437 |
| **S Soundness of interpretive approach**  **Analysis** | | |
| Is the type of analysis appropriate for the type of study?   - *thematic:* exploratory, descriptive, hypothesis generating - *framework:* e.g., policy - *constant comparison/grounded theory:* theory generating, analytical   Are the interpretations clearly presented and adequately supported by the evidence? | Analytic approach described in depth and justified  *Indicators of quality:* Description of how themes were derived from the data (inductive or deductive)  Evidence of alternative explanations being sought  Analysis and presentation of negative or deviant cases | Page 6, Line 132-137  Deviant views are presented in the results:  e.g. Page 10, Line 235-242 |
| Are quotes used and are these appropriate and effective? | Description of the basis on which quotes were chosen  Semi-quantification when appropriate  Illumination of context and/or meaning, richly detailed | Page 6, Line 134-135 |
| Was trustworthiness/reliability of the data and interpretations checked? | Method of reliability check described and justified e.g., was an audit trail, triangulation, or member checking employed? Did an independent analyst review data and contest themes? How were disagreements resolved? | Page 6, Line 132-137 |
| *Discussion and presentation* | | |
| Are findings sufficiently grounded in a theoretical or conceptual framework?  Is adequate account taken of previous knowledge and how the findings add? | Findings presented with reference to existing theoretical and empirical literature, and how they contribute | Results and discussion are presented together to facilitate comparison with the literature in an integrated way for the reader: |
| Are the limitations thoughtfully considered? | Strengths and limitations explicitly described and discussed | Page 17, Line 400-408 |
| Is the manuscript well written and accessible? | Evidence of following guidelines (format, word count)  Detail of methods or additional quotes contained in appendix  Written for a health sciences audience | Journal guidelines have been followed  Supplementary material provided to include interview questions and prompts |
| Are red flags present? These are common features of ill-conceived or poorly executed qualitative studies, are a cause for concern, and must be viewed critically. They might be fatal flaws, or they may result from lack of detail or clarity. | *Grounded theory:* not a simple content analysis but a complex, sociological, theory generating approach  *Jargon:* descriptions that are trite, pat or jargon filled should be viewed sceptically  *Over interpretation:* interpretation must be grounded in "accounts" and semi-quantified if possible or appropriate  *Seems anecdotal, self evident:* may be a superficial analysis, not rooted in conceptual framework or linked to previous knowledge, and lacking depth  *Consent process thinly discussed:* may not have met ethics requirements  *Doctor-researcher:* consider the ethical implications for patients and the bias in data collection and interpretation | N/A |

The RATS guidelines modified for BioMed Central are copyright Jocalyn Clark. They can be found in Clark JP: How to peer review a qualitative manuscript. In Peer Review in Health Sciences. Second edition. Edited by Godlee F, Jefferson T. London: BMJ Books; 2003:219-235
